# Preoperative [^68^Ga]Ga-FAPI-04 PET for evaluating pathological complete response to neoadjuvant therapy in gastrointestinal adenocarcinoma patients

**DOI:** 10.3389/fimmu.2025.1687329

**Published:** 2025-11-05

**Authors:** Xiao Zhang, Yuan Feng, Zhaoguo Lin, Ranran Chen, Yongkang Gai, Chunxia Qin, Xiaoli Lan

**Affiliations:** ^1^ Department of Nuclear Medicine, Union Hospital, Tongji Medical College, Huazhong University of Science and Technology, Wuhan, Hubei, China; ^2^ Hubei Key Laboratory of Molecular Imaging, Wuhan, Hubei, China; ^3^ Key Laboratory of Biological Targeted Therapy, The Ministry of Education, Wuhan, Hubei, China; ^4^ Department of Nuclear Medicine, Mindong Hospital, Fujian Medical University, Ningde, Fujian, China

**Keywords:** [68Ga]Ga-FAPI-04, pathologic complete response, gastrointestinal cancer, neoadjuvanttherapy, immunotherapy

## Abstract

**Objective:**

This study aimed to assess the value of preoperative [^68^Ga]Ga-FAPI-04 positron emission tomography (PET) for evaluating pathological complete response (pCR) in patients with gastrointestinal adenocarcinomas receiving neoadjuvant therapy (NAT).

**Materials and Methods:**

A retrospective analysis was conducted on patients with gastrointestinal adenocarcinomas who received [^68^Ga]Ga-FAPI-04 PET/MR scans between February 2021 and January 2024. The enrolled patients had completed preoperative NAT, undergone contemporary enhanced CT or MR scans, and received surgery within one month after PET imaging. Clinical data, imaging evaluations, PET parameters (standardized uptake values [SUVs], SUVs standardized by lean body mass [SUL], FAPI-positive tumor volume [FAPI-PTV], and total lesion burden [FAPI-TL]), and surgical pathology results were collected. Each parameter’s sensitivity, specificity, and diagnostic cutoff for predicting pCR were determined via receiver operating characteristic curve analysis. Logistic regression analysis identified independent predictors of pCR.

**Results:**

Sixty-five patients were enrolled, and 22 patients achieved pCR according to surgical pathology. In visual evaluation, [^68^Ga]Ga-FAPI-04 PET was limited in its ability to assess pCR, with 16 false positives and 1 false negative. The dichotomization using the FAPI-PTV cutoff value (<1.92 cm^3^) improved the specificity for predicting pCR to 72.7%, while retaining a high sensitivity of 93.0%. Enhanced CT or MR scans had the sensitivity and specificity of 72.7% and 93.0% in predicting pCR, respectively. According to the logistic regression analysis, a FAPI-PTV<1.92 cm^3^ was an independent predictor for patients who achieved a pCR (*p*<0.05).

**Conclusion:**

[^68^Ga]Ga-FAPI-04 PET shows promise in predicting pCR among patients with gastrointestinal adenocarcinomas following NAT. FAPI-PTV derived from [^68^Ga]Ga-FAPI-04 PET may provide an effective clinical tool for guiding further treatment.

## 1 Introduction

Gastrointestinal cancers persist as significant contributors to cancer incidence worldwide, representing more than one-fourth of cases and accounting for one-third of cancer-related mortality (1, 2). Adenocarcinomas constitute the majority of gastrointestinal cancers, accounting for more than 95.0%, and their treatment progress is highly important (1, 3). Radical resection is widely regarded as the preferred choice for treatment, but it still carries a high risk of postoperative peritoneal recurrence or metastasis (4, 5). Neoadjuvant therapy (NAT), including chemotherapy, targeted therapy, immunotherapy, and radiation therapy before radical resection, is commonly employed in patients with locally advanced or unresectable gastrointestinal cancer (6, 7).

The primary objective of NAT is to reduce tumor size, facilitate easier surgical removal, reduce micrometastatic spread, and prevent postoperative recurrence of malignant cells (8). Moreover, the increasing adoption of neoadjuvant strategies has led to the realization that pathologic complete response (pCR) is associated with favorable oncologic outcomes (9, 10). pCR refers to the absence of viable tumor cells, indicating that patients may avoid surgical intervention and postoperative complications (9, 11, 12). Recent findings have demonstrated the significant therapeutic efficacy of neoadjuvant regimens in gastrointestinal cancers, with satisfactory pCR rates of 12.9–68.8% (13, 14). Despite the significant progress made in new therapeutic strategies for gastrointestinal cancers, there are unresolved issues related to the lack of effective evaluation of pCR (15, 16). Conventional imaging techniques such as computed tomography (CT) and magnetic resonance imaging (MRI) rely primarily on morphological size, which limits their effectiveness in evaluating pathological responses in the neoadjuvant setting (17). Fluorine-18 fluorodeoxyglucose ([^18^F]FDG) positron emission tomography (PET) is widely used in various cancers and is included in the clinical guidelines (18). However, the utility of [^18^F]FDG PET in gastrointestinal adenocarcinomas is limited by physiological uptake and low metabolic activity in certain histological subtypes, particularly mucinous adenocarcinomas and signet ring cell carcinomas, which often show low uptake and reduced sensitivity for evaluating responses after NAT (19, 20). Therefore, exploring novel approaches is crucial to overcoming these limitations and enhancing the non-invasive assessment of treatment response in gastrointestinal adenocarcinoma.

Gallium-68-labeled fibroblast activation protein (FAP) inhibitor ([^68^Ga]Ga-FAPI-04) have attracted attention as promising radiotracers since 2019 owing to their superior ability to bind to FAP in the tumor stroma (21). Compared with [^18^F]FDG PET, [^68^Ga]Ga-FAPI-04 has a higher SUVmax and tumor-to-background ratio (TBR) in patients with gastric and colorectal carcinomas, thus outperforming [^18^F]FDG PET in imaging effectiveness (22, 23). Cumulative studies support the use of FAPI-PET for monitoring pathological responses in patients receiving systemic therapies, thus reinforcing its effectiveness as a clinical tool for guiding treatment (24, 25).

This retrospective study aimed to predict the pathological response to NAT via preoperative [^68^Ga]Ga-FAPI-04 PET/MR scans. Importantly, in the context of NAT, accurately predicting pCR may help guide clinical decisions, which may help prevent surgery and preserve organ function. In this study, we assessed the clinical factors and pathological findings of gastrointestinal adenocarcinoma patients, extracted PET parameters from preoperative [^68^Ga]Ga-FAPI-04 PET/MR scans and compared them with those of postoperative histopathology.

## 2 Materials and methods

### 2.1 Study patients

This retrospective study obtained approval from the Ethics Committee of our hospital (IRB number: 2020-0290). We reviewed the imaging data of all 638 patients with gastrointestinal adenocarcinoma who underwent [^68^Ga]Ga-FAPI-04 PET/MR scans between January 22, 2021, and February 8, 2024. The inclusion criteria were as follows: (1) had undergone NAT, (2) had contemporaneous preoperative enhanced CT (for gastric cancer) or enhanced MR (for colorectal cancer), (3) had postoperative pathological findings available within one month. The exclusion criteria were: (1) combined with other types of tumors; (2) incomplete clinical information; and (3) an interval of over one month between PET and the final cycle of NAT. Relevant contemporaneous clinical data, including tumor marker data and enhanced imaging results, were also collected. All pathological evaluations were independently performed by two experienced pathologists, and any discordant cases were resolved through consensus review to ensure interpretative consistency.

### 2.2 [^68^Ga]Ga-FAPI-04 PET/MR acquisition

The patients had undergone [^68^Ga]Ga-FAPI-04 PET/MR scan (SIGNA™ PET/MR; GE Healthcare, Chicago, IL, USA) after 30min post-injection (1.8–2.2 MBq/kg). PET was acquired in 3D mode, with each bed position lasting 15 minutes (DFOV = 30 cm). Simultaneously, MR imaging protocols, including T1-weighted imaging (T1WI), T2-weighted imaging (T2WI), T2-weighted fat suppression, and diffusion-weighted imaging (DWI), were executed. PET data were reconstructed using time of flight and point spread function – ordered subset expectation maximization algorithms with 28 subsets and 2 iterations, followed by a 3-mm Gaussian filter.

### 2.3 Image interpretation

Two certified nuclear medicine physicians (C.Q. with 15 years and X.Z. with 7 years of experience in nuclear medicine) and a radiologist (F.L. with 20 years of experience in radiology and 8 years in nuclear medicine) independently reviewed the images with access to clinical data while blinded to the patients’ pathology results, making diagnoses on the basis of the criteria outlined below. In cases of divergence, a consensus diagnosis was reached through discussion.

CT evaluation for gastric cancer included assessments of tumor thickness, CT attenuation, and lymph node diameter on post-NAT imaging for gastric cancer (26). MRI evaluations for colorectal cancer have focused on the T- and N-stages, extramural venous invasion, mesorectal fascia invasion, and tumor location for patients with colorectal cancer (27).

For PET imaging, visual analysis classified lesions as positive if their [^68^Ga]Ga-FAPI-04 activity surpassed that of nearby background tissues. The quantitative PET parameters were obtained via the automated PET VCAR (Volume Computer Assisted Reading) segmentation software system from the Advantage Workstation (version AW4.6, GE Healthcare) (28). Lesion volumes of interest were delineated using the AI-driven Auto Contour tool. Using PET VCAR, the software automatically calculated the maximum standardized uptake value (SUVmax), SUV normalized to lean body mass (SUL), FAPI-positive tumor volume (FAPI-PTV), and total lesion FAP expression (FAPI-TL).

### 2.4 Statistical analysis

Statistical analysis was conducted via SPSS, version 22 (IBM, Armonk, NY, USA). Continuous variables are presented as the means ± standard deviations and were assessed with the *t*-test. Categorical variables were evaluated through the chi-square test. Receiver operating characteristic (ROC) curve analyses were used to evaluate the area under the curve (AUC), sensitivity, specificity, and diagnostic cutoff values of each variable. Logistic regression was used to obtain an independent predictor of pCR. Two-tailed *p* values of less than 0.05 were regarded as statistically significant.

## 3 Results

### 3.1 Patient characteristics

**Figure 1:**
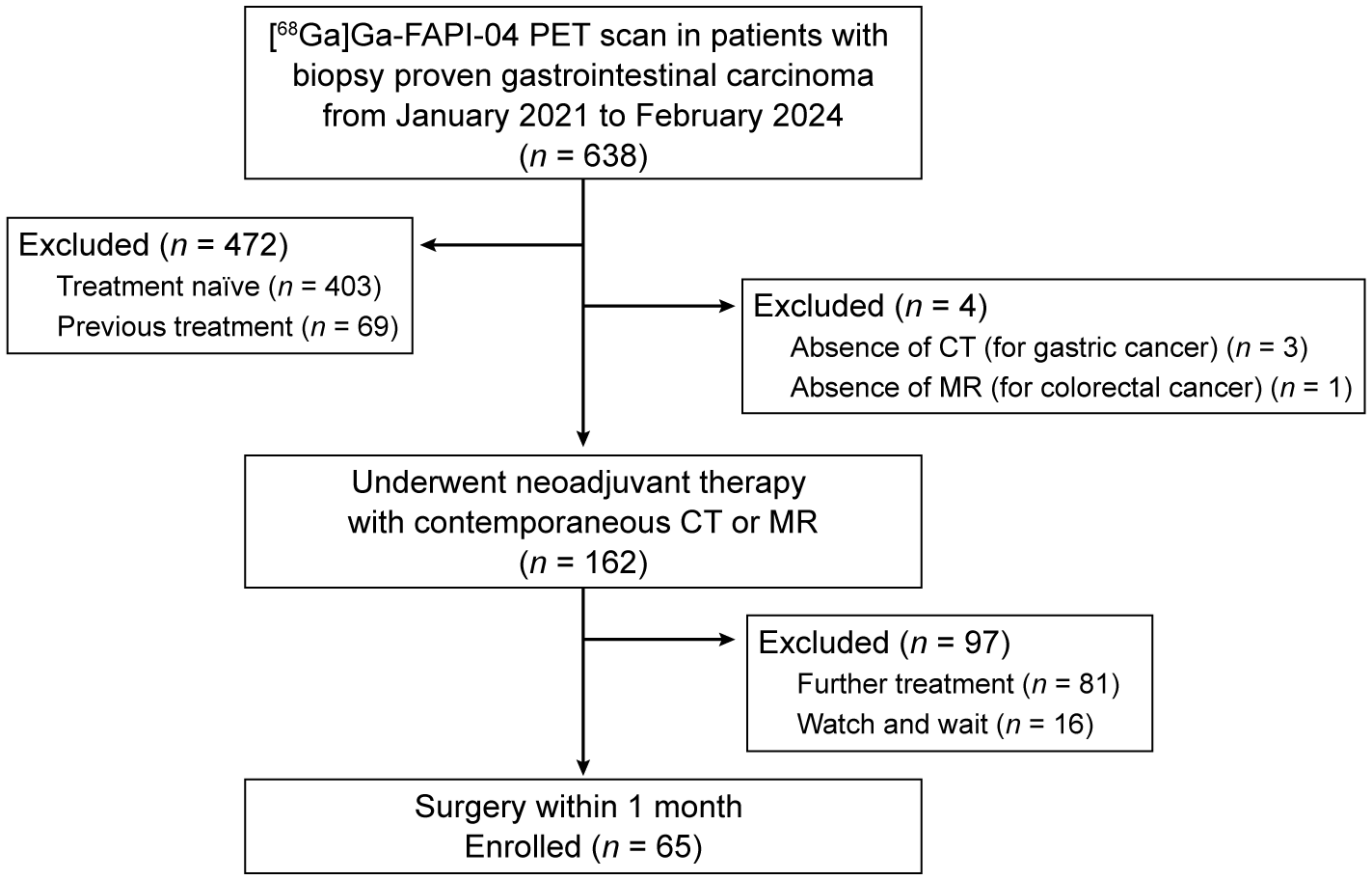
Trial profile.

A total of sixty-five patients with gastrointestinal adenocarcinoma (38 men and 27 women), aged between 27 and 75 years, were ultimately included in this study (**Figure 1**). Among them, 25 patients had gastric adenocarcinomas, 11 had colonic adenocarcinomas, and 29 had rectal adenocarcinomas (**Table 1**). One patient had elevated serum levels of carbohydrate antigen 19-9 (CA19-9), carcinoembryonic antigen (CEA), and carbohydrate antigen 72-4 (CA72-4), and three had elevated levels of CA19–9 and CEA. One patient had an elevated CEA level only, and another had an elevated CA19–9 level. Regarding treatment regimens, almost all patients (63/65, 96.9%) received chemotherapy, either alone (21, 32.3%) or in combination with other modalities. Specifically, 36 patients (55.4%) underwent combined immunochemotherapy, among whom 24 also received concurrent radiotherapy. Smaller subsets of patients received chemotherapy with targeted therapy (3, 4.6%), chemotherapy with radiotherapy (2, 3.1%), or a combination of chemotherapy, immunotherapy, and targeted therapy (3, 4.6%). Only one patient each received immunotherapy alone (1.5%) or targeted therapy alone (1.5%).

**Table 1:** Patient characteristics.

Characteristics	Patients (*n* = 65)
Age, years, mean ± SD (range)	57.72 ± 10.04 (27–75)
Sex, *n* (%)	
Male	38 (58.46)
Female	27 (41.54)
ECOG performance status, *n* (%)	
0	26 (40.00)
1	37 (56.92)
2	2 (3.08)
Tumor types, *n* (%)	
Gastric adenocarcinoma	25 (38.46)
Colonic adenocarcinoma	11 (16.92)
Rectal adenocarcinoma	29 (44.62)
Serum tumor marker level, *n* (%)	
Normal	59 (90.77)
Abnormal CEA level (≥5 ng/mL)	5 (7.69)
Abnormal CA19–9 level (≥37.0 U/L)	5 (7.69)
Abnormal CA72–4 level (≥6.9 U/L)	1(1.54)
Serum LDH level (109–245, U/L)	194.74 ± 47.31
Treatment, *n* (%)	
Chemotherapy	21 (32.31)
Immunotherapy	1 (1.54)
Targeted therapy	1 (1.54)
Chemotherapy and immunotherapy	8 (12.31)
Chemotherapy and targeted therapy	3 (4.62)
Chemotherapy and radiation treatment	2 (3.08)
Chemotherapy, immunotherapy, and targeted therapy	3 (4.62)
Chemotherapy, immunotherapy, and radiation treatment	24 (36.92)
Chemotherapy, targeted therapy, and radiation treatment	2 (3.08)
Pathological disease stage, *n* (%)	
pCR	22 (33.85)
Stage I	10 (15.38)
Stage II	11 (16.92)
Stage III	16 (24.62)
Stage IV	6 (9.23)

SD, standard deviation; ECOG, Eastern Cooperative Oncology Group; CEA, carcinoembryonic antigen; CA, carbohydrate antigen; LDH, lactate dehydrogenase; pCR, pathologic complete response.

### 3.2 PET visual analysis and surgery

PET visual analysis revealed positive [^68^Ga]Ga-FAPI-04 uptake in 58 patients, whereas 7 patients had negative results. Among those patients, the pCR rate was 33.85% (22/65), as determined by subsequent surgical pathology. For non-pCR patients, surgical pathology confirmed a total of 117 malignant lesions, 80 of which were detected via [^68^Ga]Ga-FAPI-04 PET. **Supplementary Table S1** displayed lesion-based diagnostic performance of PET visual analysis. The percentages of patients with pathological stages I, II, III, and IV disease were 15.38% (10/65), 16.92% (11/65), 24.62% (16/65), and 9.23% (6/65), respectively.

### 3.3 Clinical and PET characteristics according to pCR

**Figure 2:**
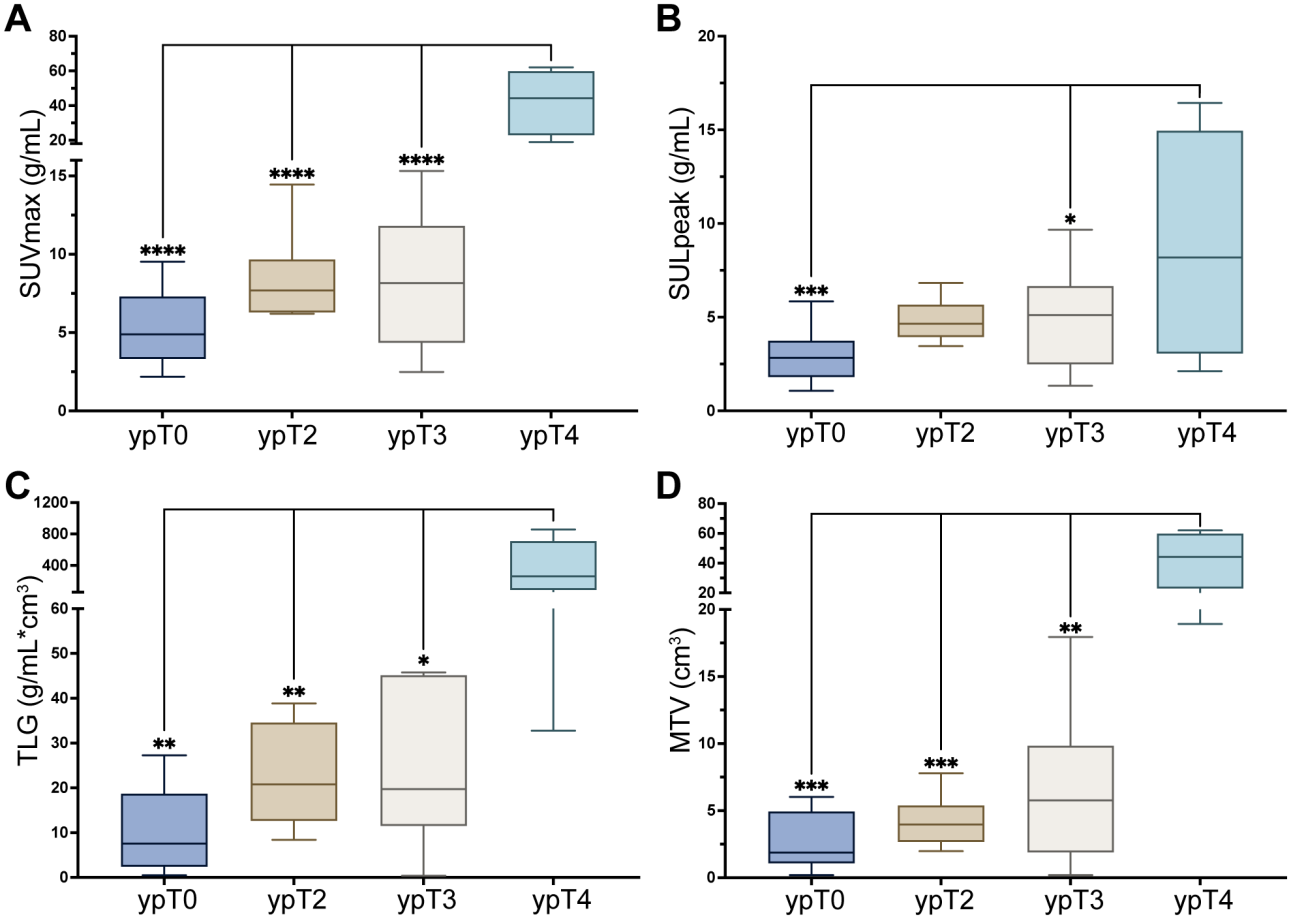
**(A**–**D)** [^68^Ga]Ga-FAPI-04 PET parameters in different T staging. All the data were compared with pT4. *p<0.05, **p<0.01, ***p<0.001, ***p<0.0001.

Clinical characteristics, including age, LDH levels, and treatment regimens, were analyzed alongside PET characteristics to identify the factors influencing the pathological response (**Table 2**). Correlation analysis of PET parameters was shown in **Supplementary Figure S1**. PET parameters, specifically the FAPI-PTV and FAPI-TL, were significantly lower in the pCR group than in the non-pCR group (*p* = 0.01). Patients with ypT4 disease had significantly greater SUVmax, SULpeak, FAPI-PTV, and FAPI-TL values than those with ypT0, T2, and T3 disease (*p*<0.05, **Figure 2**).

**Table 2:** Patient-based analyses according to pCR.

Characteristics	Patients with pCR	Patients with non-pCR	*p* value
Age (mean ± SD, years)	58.77 ± 10.09	57.19 ± 10.09	0.55
LDH level (109–245, U/L)	192.68 ± 42.44	195.79 ± 50.06	0.80
Primary tumor site, *n* (%)	22 (33.85)	43 (66.15)	0.37^
Gastric adenocarcinoma	8 (12.31)	17 (26.15)	
Colonic adenocarcinoma	2 (3.08)	9 (13.85)	
Rectal adenocarcinoma	12 (18.46)	17 (26.15)	
Treatment, *n* (%)	22 (33.85)	43 (66.15)	
Chemotherapy	21 (32.31)/1 (1.54)	42 (64.61)/1 (1.5)	1.0^^
Immunotherapy	15 (23.08)/7 (10.77)	21 (32.31)/22 (33.85)	0.14
Targeted therapy	2 (3.08)/20 (30.77)	7 (10.77)/36 (55.38)	0.71^^
Radiation treatment	12 (18.46)/10 (15.38)	16 (24.62)/27 (41.54)	0.18
PET (*n* = 58)	*n* = 16	*n* = 42	
SUVmax	6.01 ± 3.07	8.54 ± 4.91	0.06
SULpeak	3.49 ± 1.72	5.03 ± 3.04	0.06
FAPI-PTV	4.69 ± 7.41	17.20 ± 25.45	0.01*
FAPI-TL	20.43 ± 34.69	100.56 ± 192.36	0.01*

^ *p* value was calculated by using the likelihood ratio test.

^^ *p* values were calculated by using the Fisher-Freeman-Halton exact test.

* *p* values were calculated by Welch’s *t*-test.

### 3.4 The predictive efficacy of [^68^Ga]Ga-FAPI-04 PET for pCR

**Figure 3:**
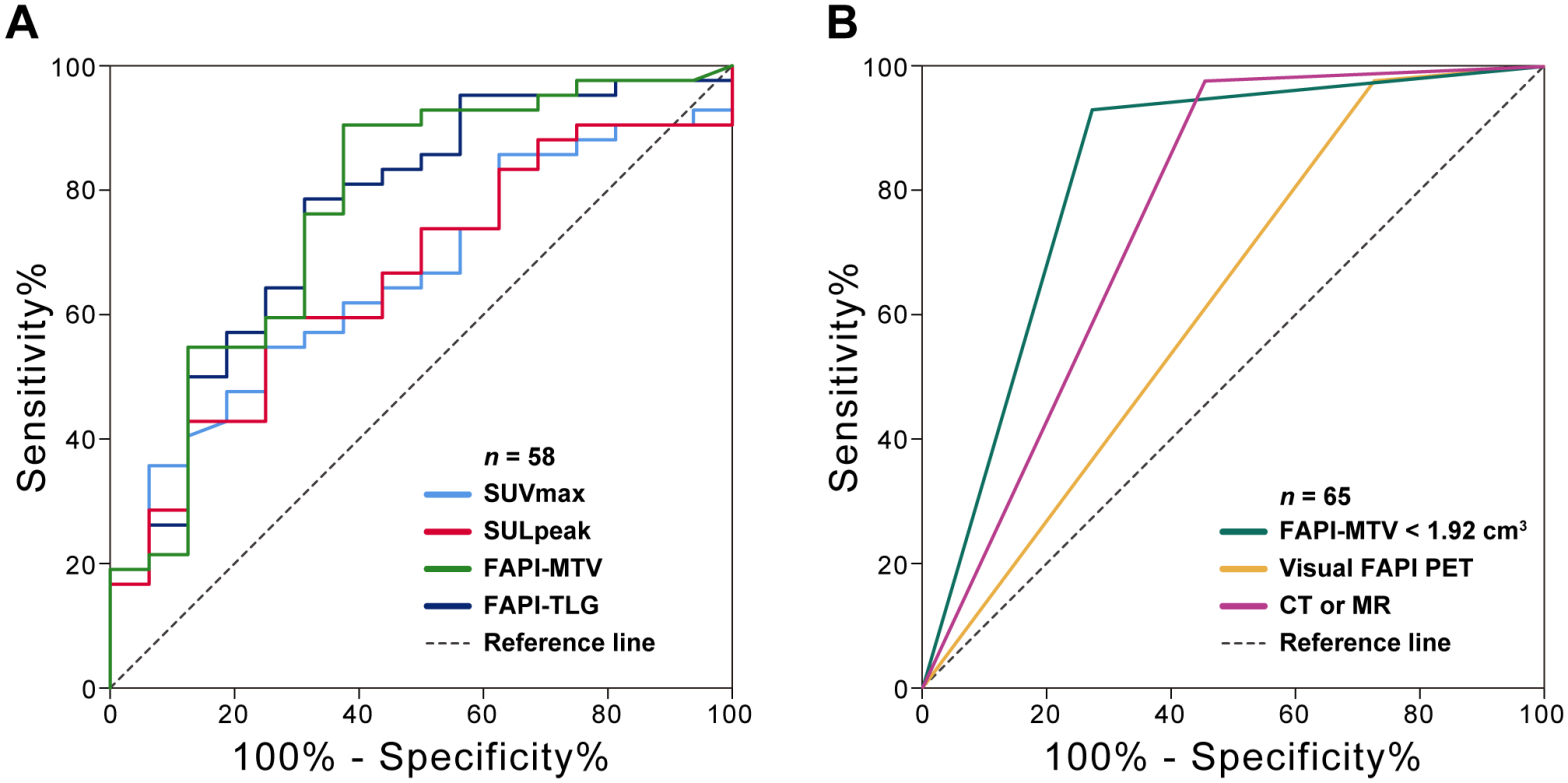
ROC curves of the ability of [^68^Ga]Ga-FAPI-04 PET and CT or MR enhanced scan to predict pCR. **(A)** Quantitative FAPI-PET parameters to predict pCR (*n* = 58); **(B)** Visual FAPI PET, quantitative FAPI-PTV (<1.92 cm^3^) and contemporaneous CT or MR to predict pCR (*n* = 65).

Among the quantitative PET parameters for evaluating pCR in 58 patients with FAPI-positive findings, the FAPI-PTV had the largest AUC (0.77; **Figure 3A** and **Table 3**). Compared with visual evaluation, optimal dichotomization using the FAPI-PTV cutoff value (<1.92 cm^3^) in all 65 patients (**Figure 3B**) resulted in increased specificity (72.73%) but high sensitivity (93.02%, *p*<0.05). The accuracy of FAPI-PTV<1.92 cm^3^ for assessing pCR in gastric and colorectal adenocarcinoma is 84.0% and 87.50%, respectively. Compared with PET visual evaluation, contrast-enhanced CT or MR scans had an accuracy of 83.07% in predicting pCR, but this difference was not significant. The detailed diagnostic performance of FAPI-PTV<1.92 cm^3^ and CT/MR validation was provided in **Table 4**. Additionally, a logistic regression model was performed, which included PET parameters (SUVmax, SULpeak, FAPI-PTV, and FAPI-TL), visual assessment, and FAPI-PTV dichotomization (<1.92 cm^3^). The analysis revealed that a FAPI-PTV<1.92 cm^3^ was an independent predictor of pCR in all patients (**Supplementary Table S2**).

**Table 3:** Predictive performance of visual and quantitative PET assessment for pCR.

Categorization	Parameters	AUC	*p* value	95%CI	Accuracy	Sensitivity	Specificity
Visual positive on FAPI PET (*n* = 58)	FAPI-PTV	0.77	0.002	0.63–0.91	82.76%	62.50%	90.48%
All patients (*n* = 65)	Visual	0.62	0.10	0.47–0.78	73.85%	27.27%	97.67%
	FAPI-PTV<1.92 cm^3^	0.83	<0.001	0.71–0.95	86.15%	72.73%^	93.02%
	CT or MR	0.76	0.001	0.62–0.90	83.07%	54.54%	97.67%
Gastric* (*n* = 25)	FAPI-PTV<1.92 cm^3^	0.82	0.012	0.62–1.00	84.00%	75.00%	88.24%
Colorectal* (*n* = 40)	FAPI-PTV<1.92 cm^3^	0.84	<0.001	0.69–0.99	87.50%	71.42%	96.15%

AUC, area under curve; CI, confidence interval.

^*p* value<0.05 compared with visual assessment.

*Only listed the best performance parameter.

**Table 4:** Patient-based diagnostic performance for pCR.

Pathology Parameter		Non-pCR	pCR	Total
FAPI-PTV	>1.92 cm^3^	40	6	46
	<1.92 cm^3^ + PET (–)	3	16	19
CT or MR	Positive	42	10	52
	Negative	1	12	13
Total		43	22	65

### 3.5 Typical cases

**Figure 4:**
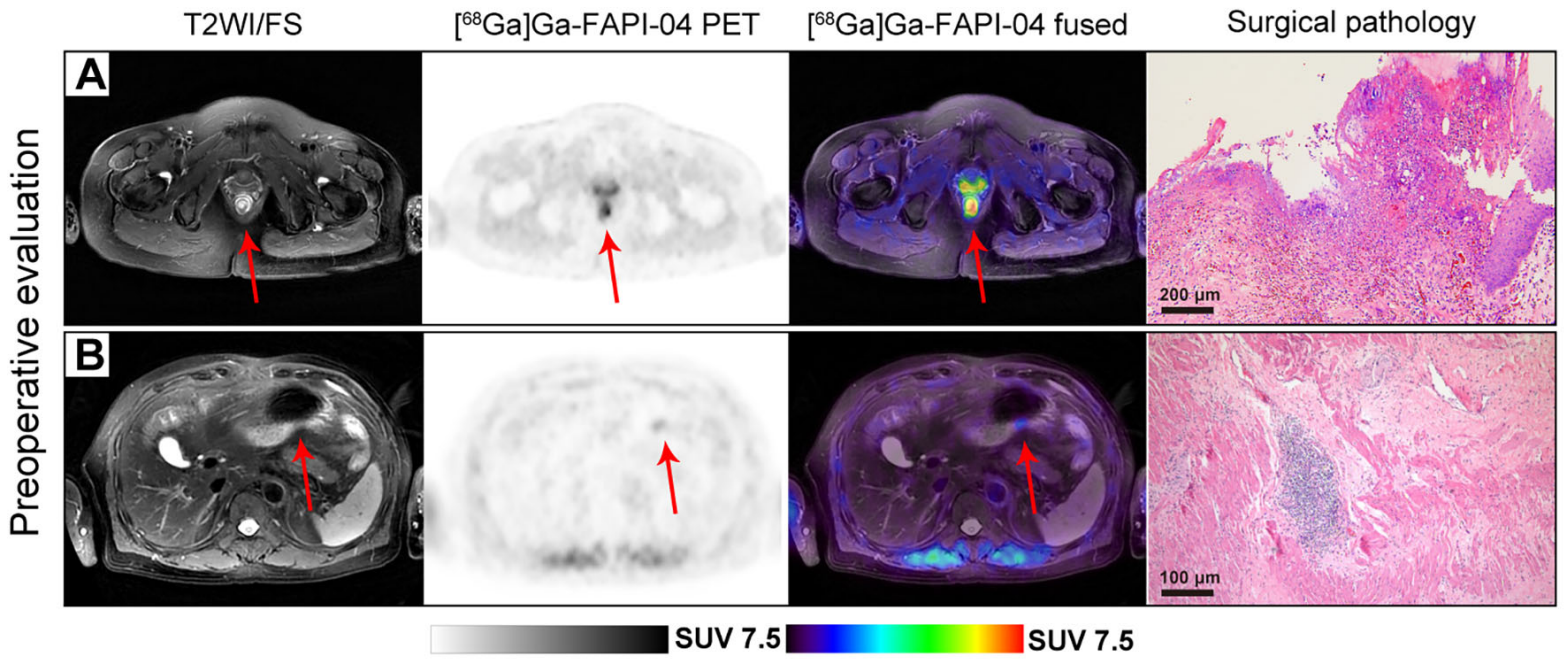
**(A)** A patient in her 50s presented with a 2-month history of hematochezia, which led to the diagnosis of adenocarcinoma in the lower rectum. Following neoadjuvant short-course radiotherapy combined with immunochemotherapy, [^68^Ga]Ga-FAPI-04 PET/MR revealed avid tracer accumulation (SUVmax 6.8, SULpeak 3.76, FAPI-PTV 1.41 cm^3^, FAPI-TL 7.56, indicated by red arrows) in the lower rectum. However, subsequent surgical pathology revealed no residual tumor cells (pCR), although a chronic ulcer with hyperplasia of granulation tissue was observed. **(B)** In another case, a 75-year-old man underwent chemotherapy for gastric adenocarcinoma. A slight focal uptake (SUVmax 2.18, SULpeak 1.75, FAPI-PTV 1.87 cm^3^, FAPI-TL 2.35) was observed in [^68^Ga]Ga-FAPI-04 PET/MR imaging, but further surgical pathology confirmed the lesion as pCR.

Focal uptake was visually detected in the preoperative [^68^Ga]Ga-FAPI-04 PET/MR scans of patients with rectal and gastric cancer (**Figure 4**), respectively. However, subsequent surgical pathology indicated pCR in both patients. The FAPI-PTVs of the patients were 1.41 cm^3^ and 1.87 cm^3^, respectively, which were below the cutoff values (1.92 cm^3^), indicating pCR.

**Figure 5:**
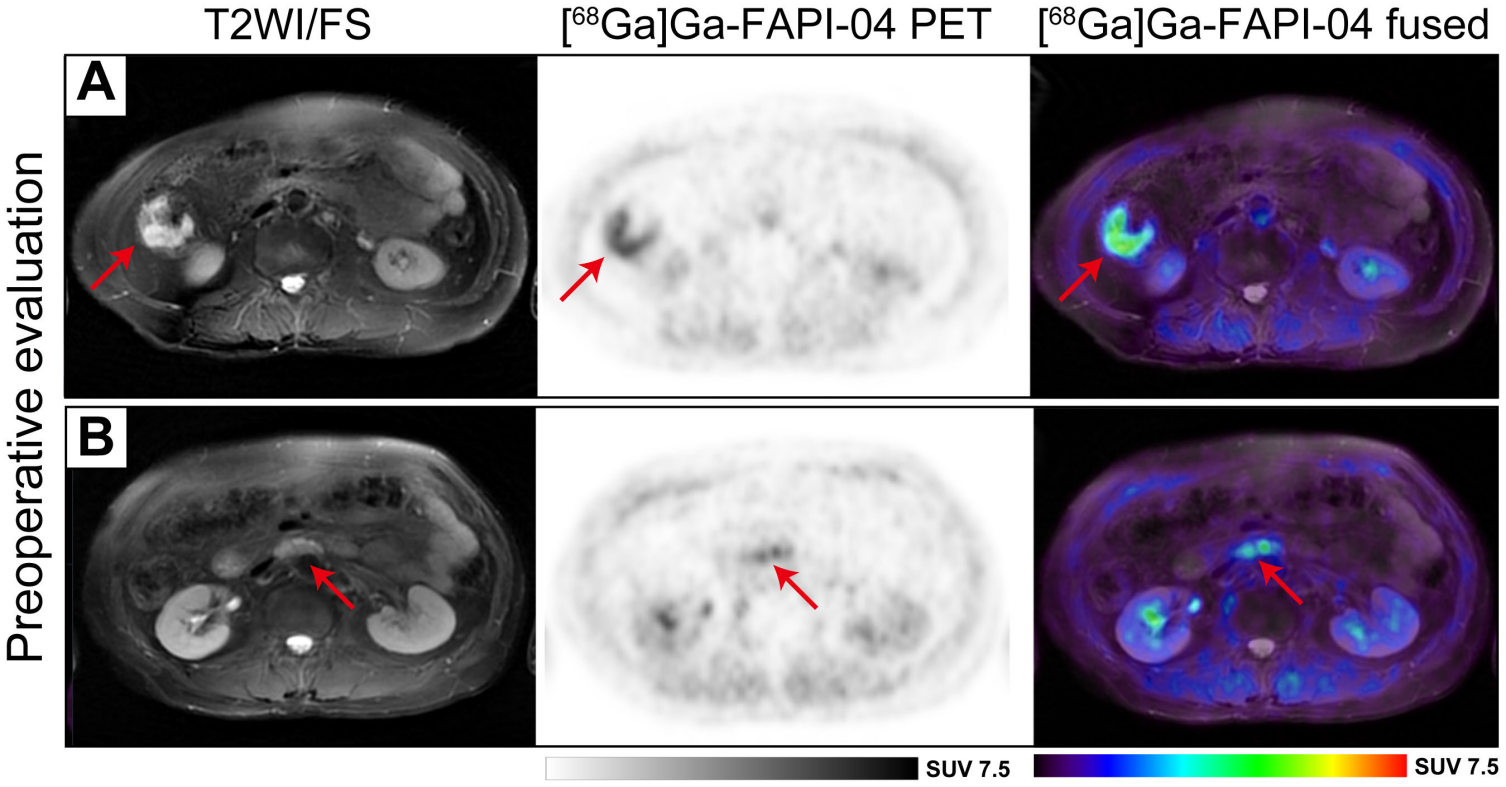
A 59-year-old male patient presented with black stool, anemia, and elevated levels of CEA and CA19-9. A colonoscopy biopsy confirmed adenocarcinoma of the ascending colon, leading to subsequent neoadjuvant immunotherapy. **(A, B)** [^68^Ga]Ga-FAPI-04 PET/MR revealed avid tracer accumulations (indicated by red arrows) in the ascending colon (SUVmax 4.79, SULpeak 2.66, FAPI-PTV 14.09 cm^3^, FAPI-TL 37.82) and retroperitoneal lymph node (SUVmax 4.69, SULpeak 2.49, FAPI-PTV 6.03 cm^3^, FAPI-TL 13.54). Subsequent surgical pathology revealed no residual tumor cells in these lesions.

A 59-year-old male patient has undergone immunotherapy. The preoperative [^68^Ga]Ga-FAPI-04 PET/MR scan revealed thickening of the ascending colon wall and an enlarged lymph node with increased uptake (**Figure 5**). The FAPI-PTV values of the lesions were 14.09 and 6.03 cm^3^, respectively, further surgical pathology indicated false-positive.

## 4 Discussion

Accurate evaluation of the pathological response of patients after neoadjuvant therapy is crucial for treatment decisions, especially as it may reduce surgical intervention for patients. Our findings indicate that visual analysis of [^68^Ga]Ga-FAPI-04 PET is limited in its ability to predict pCR. However, when the FAPI-PTV cutoff value of 1.92 cm^3^ was used, the diagnostic efficiency of [^68^Ga]Ga-FAPI-04 PET for pCR significantly improved. To the best of our knowledge, this study represents the initial demonstration of the ability of visual qualitative and quantitative features derived from [^68^Ga]Ga-FAPI-04 PET scans to predict pCR in gastrointestinal adenocarcinomas within the emerging neoadjuvant context.

Efficient imaging methods to assess pCR after NAT are essential for supporting presurgical clinical decision-making. A comprehensive overview of large studies underscores the impressive diagnostic performance of FAPI-PET in identifying primary or peritoneal metastatic lesions in gastrointestinal cancers (19, 24, 29). Qin et al. analyzed the cellular dynamics of 29 patients who underwent NAT via single-cell and spatial transcriptome sequencing (30). In patients who achieved complete or near-complete regression, the FAP^+^CAF (FAP-positive CAF) subset in surgical samples decreased to minimal levels following NAT, which enables the feasibility of FAPI-PET to assess pCR. Backhaus et al. reported that visual evaluation via [^68^Ga]Ga-FAPI-46 PET/MR accurately evaluated the pathologic response status of a small group (13 patients) of breast cancer patients after neoadjuvant chemotherapy (31). However, in our study, visual assessment of [^68^Ga]Ga-FAPI-04 PET remained limited in predicting pCR after NAT, as evidenced by 16 patients with false-positive [^68^Ga]Ga-FAPI-04 uptake. Several factors contribute to this limitation. Among these patients, 11 (68.75%) had received radiotherapy, which can induce local fibrosis. Such changes may lead to sustained FAP expression, as activated fibroblasts around the lesions can remain metabolically active longer than tumor cells, thereby producing increased uptake and false-positive signals (32). In the remaining cases, false-positive findings may have been associated with inflammatory cell infiltration following immunotherapy, targeted therapy, chemotherapy, or combined regimens (33, 34). Moreover, physiological [^68^Ga]Ga-FAPI-04 uptake in certain normal tissues may also contribute to false-positive results, potentially reducing the accuracy of preoperative evaluation. Furthermore, one patient exhibited negative [^68^Ga]Ga-FAPI-04 uptake, possibly due to a small lesion volume beyond PET scanner detection (34, 35). All of these factors point to a potential pitfall of FAPI-PET visual evaluation in accurately predicting the efficacy of NAT.

Notably, certain quantitative [^68^Ga]Ga-FAPI-04 PET parameters demonstrated the ability to distinguish patients who achieved a pCR, with the FAPI-PTV displaying the largest AUC. Additionally, a FAPI-PTV<1.92 cm^3^ was more effective at predicting pCR. A lower FAPI-PTV signifies a reduced tumor burden with a small FAP-active lesion among the total lesions, indicating a greater likelihood of achieving pCR after NAT (29, 36). Miao et al. examined the value of quantitative analysis (SUVmax, SUVpeak, and TBR values) from FAPI-PET in predicting the pathologic response in a neoadjuvant scenario (25). More PET parameters were included in this study, and both univariate and multivariate testing indicated that the FAPI-PTV from [^68^Ga]Ga-FAPI-04 PET scans could be a more effective predictor for pCR. Similarly, Nicolas et al. evaluated survival outcomes in 212 rectal cancer patients treated with standard neoadjuvant chemoradiotherapy and surgery (37). They underscored the pivotal role of CAFs in influencing tumor behavior and treatment response. Given the quantitative evaluation of FAP^+^CAFs, [^68^Ga]Ga-FAPI-04 PET imaging could provide valuable insight into stromal remodeling after NAT (38). Integrating FAP-targeted PET imaging could improve therapeutic assessment, offering a biologically informed method for identifying patients who may avoid surgery and related complications.

In our study, different imaging modalities were also applied to compare, demonstrating limited predictive benefit of CT and MR. Previous studies have shown that preoperative CT lacks accuracy in predicting the response to NAT in patients with gastric cancer (39). Similarly, MR imaging has proven unreliable for the preoperative staging of rectal cancer patients with tumors less than 5 mm in thickness (40). Furthermore, these modalities are limited in distinguishing necrotic tumor tissue from residual lesions, particularly in the context of emerging neoadjuvant immunotherapy (41, 42).

There were several limitations in our study. First, the diversity in neoadjuvant regimens might have introduced bias. Second, due to the retrospective design of the study, a multicenter and prospective clinical trial involving a larger cohort of patients with gastrointestinal adenocarcinoma undergoing NAT is warranted to address these limitations and comprehensively validate our results. Additionally, integrating complementary biomarkers, such as circulating tumor DNA, could enhance the precision of response assessments and provide a more comprehensive view of tumor dynamics.

Our study suggests that [^68^Ga]Ga-FAPI-04 PET imaging can be used to evaluate pCR after NAT. The optimal FAPI-PTV cutoff value of 1.92 cm^3^ could serve as a reliable predictor of pCR in patients with gastrointestinal tumors in the evolving neoadjuvant setting.

## Data Availability

The raw data supporting the conclusions of this article will be made available by the authors, without undue reservation.
