# Supplementary material for: Preoperative [68Ga]Ga-FAPI-04 PET for evaluating pathological complete response to neoadjuvant therapy in gastrointestinal adenocarcinoma patients
Source: Front Immunol. 2025 Nov 5;16:1687329. doi: 10.3389/fimmu.2025.1687329 (PMC12627024; doi:10.3389/fimmu.2025.1687329)
Supplement: Supplementary file 1 [file DataSheet1.docx]

Supplementary Material

For

**Preoperative [^68^Ga]Ga-FAPI-04 PET for evaluating pathological complete response to neoadjuvant therapy in gastrointestinal adenocarcinoma patients**

# Supplementary Figures and Tables

## Supplementary Figures

**
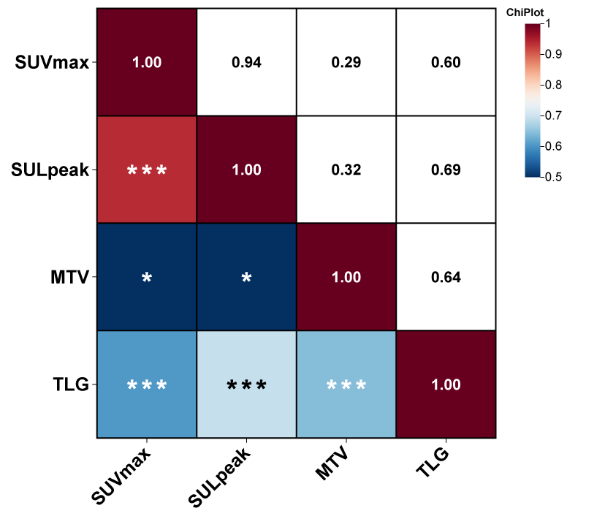
**

**Figure S1.** Correlation among quantitative PET parameters. Pairwise correlations between SUVmax, SULpeak, FAPI-PTV, and FAPI-LG were assessed to evaluate potential collinearity.

## Supplementary Tables

Surgical pathology confirmed a total of 117 malignant lesions in 65 patients, 80 of which were detected via [^68^Ga]Ga-FAPI-04 PET. The lesion-based diagnostic performance is shown in Table S1.

| Table S1 Lesion-based Diagnostic Performance of PET Visual Analysis | | | |
| --- | --- | --- | --- |
| Pathology  PET | **Positive** | **Negative** | **Total** |
| Positive | 80 | 22 | 102 |
| Negative | 37 | 758 | 795 |
| Total | 117 | 780 | 897 |

A logistic regression model was performed, which included PET parameters (SUVmax, SULpeak, FAPI-PTV, and FAPI-TL), visual assessment, and FAPI-PTV dichotomization (<1.92 cm^3^). The analysis revealed that a FAPI-PTV<1.92 cm^3^ was an independent predictor of pCR in all patients (**Table S2**).

| **Table S2 Logistic Regression Analysis for Predicting pCR** | | | |
| --- | --- | --- | --- |
| **Variable** | **pCR** | | |
|  | B | *P* value | Exp (B) |
| **FAPI-PTV<1.92 cm^3^** | 3.21 | **0.003** | 24.85 |
| **SUVmax** | -0.91 | 0.70 | 0.91 |
| **SULpeak** | 0.19 | 0.67 | 1.21 |
| **FAPI-PTV** | -0.02 | 0.75 | 0.98 |
| **FAPI-TL** | -0.002 | 0.84 | 1.00 |
| **CT or MR** | 22.15 | 1.00 | / |
